# Physiological Effect of Deep Pressure in Reducing Anxiety of Children with ASD during Traveling: A Public Transportation Setting

**DOI:** 10.3390/bioengineering9040157

**Published:** 2022-04-05

**Authors:** Ilham Yustar Afif, Aloysius Raynaldo Manik, Kristian Munthe, Mohamad Izzur Maula, Muhammad Imam Ammarullah, Jamari Jamari, Tri Indah Winarni

**Affiliations:** 1 Undip Biomechanics Engineering & Research Centre (UBM-ERC), Diponegoro University, Semarang 50275, Central Java, Indonesia; iyustar.afif@gmail.com (I.Y.A.); izzurmaula@gmail.com (M.I.M.); imamammarullah@gmail.com (M.I.A.); j.jamari@gmail.com (J.J.); 2 Department of Mechanical Engineering, Faculty of Engineering, Diponegoro University, Semarang 50275, Central Java, Indonesia; aloysiusmanik06@gmail.com (A.R.M.); kristian.munthe1995@gmail.com (K.M.); 3 Department of Anatomy, Faculty of Medicine, Diponegoro University, Semarang 50275, Central Java, Indonesia; 4 Center for Biomedical Research (CEBIOR), Faculty of Medicine, Diponegoro University, Semarang 50275, Central Java, Indonesia

**Keywords:** autism spectrum disorder, arousal, traveling, deep pressure, heart rate, skin conductance

## Abstract

Traveling with children with autism can be very challenging for parents due to their reactions to sensory stimuli resulting in behavioral problems, which lead to self-injury and danger for themselves and others. Deep pressure was reported to have a calming effect on people with autism. This study was designed to investigate the physiological effect of deep pressure, which is an autism hug machine portable seat (AHMPS) in children with autism spectrum disorders (ASD) in public transportation settings. The study was conducted with 20 children with ASD (16 boys and 4 girls) at the Semarang Public Special School with an age ranging from 4 to 13 years (mean 10.9 ± 2.26 years), who were randomly assigned into two groups. The experiment consisted of group I who used the AHMPS inflatable wraps model and group II who used the AHMPS manual pull model. Heart rate (HR) and skin conductance (SC) were analyzed to measure the physiological calming effect using pulse oximeter oximetry and a galvanic skin response (GSR) sensor. Heart rate was significantly decreased during the treatment compared to the baseline (pre-test) session in group I (inflating wrap model) with *p* = 0.019, while no change of heart rate variability (HRV) was found in group II (manual pull model) with *p* = 0.111. There was no remaining effect of deep pressure using the HRV indicator after the treatment in both groups (group I with *p* = 0.159 and group II with *p* = 0.566). GSR captured the significant decrease in skin conductance during the treatment with *p* < 0.0001 in group I, but no significant decrease was recorded in group II with *p* = 0.062. A skin conductance indicator captured the remaining effect of deep pressure (after the treatment); it was better in group I (*p* = 0.003) than in group II (*p* = 0.773). In conclusion, the deep pressure of the AHMPS inflating wrap decreases physiological arousal in children with ASD during traveling.

## 1. Introduction

Autism Spectrum Disorder (ASD) refers to a group of complex neurodevelopmental disorders that begin in the developmental period [1] and is indicated by deficits in communication and social interaction as well as restricted and repetitive interest behaviors that are usually recognized in early childhood with the presence of two major symptoms: social–communication deficits and restricted and repetitive interests/behaviors [2]. The number of individuals with ASD is increasing, especially in Indonesia. Data from the Ministry of Women’s Empowerment and Child Protection of the Republic of Indonesia stated that in 2018, the population was around 2.4 million with increased cases of around 500 new ASD cases per year [3]. The pathophysiological mechanism of autism is still elusive, but several factors are believed to contribute to pathogenesis. ASD is now known to involve complex interaction between genetics and environment, with heritability ranging from 40 to 80% [4]. Several genes have been implicated in the pathogenesis of ASD, with most being involved in synaptic dysfunction. Several environmental factors and associated conditions have been linked to the mechanism, such as immune dysfunction, prenatal and perinatal factors, socio-economic status, advanced parental age, and drug or toxic exposure [5].

Most people with ASD have sensory processing disorder (SPD), which is an inability to respond behavioral response to sensory input experienced due to impaired sensory input such as sound, touch, body movement, sight, taste, and smell, with the prevalence of SPD in autism estimated to be around 90% [6]. Atypical reactions to sensory stimuli in children with autism can affect their daily life activity performances and enjoyment tasks [7,8]. Thus, it can be very challenging for parents when traveling with children with autism due to reactions to sensory stimuli resulting in behavioral problems, e.g., crying, screaming, shouting, kicking, banging toys, and throwing, which lead to self-injury and danger to themselves and others, including the driver [9]. In a survey of 703 adults with ASD in New Jersey regarding their travel experience, 291 had sensory issues [10]. Excessive sensory stimuli such as loud noises, unfamiliar smells, the touch of others such as security manual body searches, and spontaneous, unplanned, and highly stimulating activities during traveling trigger reactions to sensory stimuli [11]. Therefore, parents, caregivers, or people accompanying children with ASD on a trip have to prepare, for example, when dealing with noise during a trip, one can use noise-canceling earphones to provide relief [12].

In order to alleviate the symptoms of ASD, many treatments have been developed, such as behavioral treatment, psychopharmacological treatment, speech and language treatment, occupational treatment, social skills training, and complementary treatment [13]. Based on the previous study, people in low–middle-income countries tend to choose the fastest, easiest, and most cost-effective treatment [14]; one of these was sensory integration therapy (part of complementary treatment) [13,15]. Deep pressure, which is an application of sensory integration therapy, is able to provide a calming effect and reduce unwanted movements due to anxiety, obtained by applying pressure to the body at a certain time [16,17]. When the body receives deep pressure, this sensory information modulates the autonomic nervous system to produce a calming effect [18,19]. Grandin introduced his own experimental tool using the deep pressure method called the ‘Squeeze Machine’. The device has a V pad which is used as pressure exertion on the body and provides comfort to the wearer [20].

Several studies that have been conducted to investigate deep pressure devices (e.g., squeeze machines, weighted blankets, and pressure vests) have reported positive effects after the use of these devices. Edelson et al. [21] evaluated Grandin’s squeeze machine on 12 children with autism randomly assigned to two groups (experimental and placebo groups) and then reported that administering deep pressure had a calming effect or benefit for children with autism with high levels of anxiety or arousal. The administration of deep pressure treatment with a 30-pound weighted blanket displayed no adverse effects on the user’s vital signs (blood pressure, pulse rate, and pulse oximetry) targeting adult mental health consumers [22,23]. Research related to the effect of using a pressure vest has been conducted. This study tested the effect of a pressure vest in the form of a Vayu vest on 50 healthy adults, where the pressure from the Vayu vest was given by the participants themselves until they felt comfortable, and then, stress was induced through the Moron Test. The results of these treatments showed that the use of a pressure vest can affect the activity of the autonomic system and provide a calming effect on the participants [24]. The calming effect and anxiety reduction were shown in the provision of deep pressure treatment in the form of a weighted blanket under wisdom tooth extraction [25] and third molar extraction conditions [26].

A type of device that applies deep pressure theory is the autism hug machine portable seat (AHMPS), which is a seat-like device made of soft foam and semi-leather material and has two horizontal hug parts located in the chest and thigh to provide safety as well as deep pressure stimulation. Previous studies were conducted to investigate the effect of a deep pressure device called the autism hug machine portable seat (AHMPS) in reducing behavioral and biological arousal in children with ASD [27,28], but these studies were still limited to being carried out in the classroom, not in traveling situations. Therefore, this study was designed to investigate the effect of deep pressure, is the AHMPS, in children with ASD in public transportation settings. This study aimed to compare the effectiveness of the deep pressure application of the AHMPS inflatable wrap and manual pull model in children with ASD in a public transportation (bus) setting, observed from physiological response, i.e., heart rate variability, and skin conductivity.

## 2. Materials and Methods

### 2.1. Study Design and Participants

This study was an experimental study with a repeated-measures design to investigate the effect of deep pressure on public transportation. Twenty children with ASD from Semarang Public Special School with an age ranging from 4 to 13 years, who were randomly assigned into two experimental groups with different AHPMS models, were included in this study. Prior to the study, all children who were matched with the inclusion criteria were familiarized with the devices and experimental procedures, including the presence of researchers. Only children who agreed to participate in this study were included. The two groups consisted of group I who used the AHMPS inflatable wraps model and group II who used the AHMPS manual pull model. The treatment was administered to two groups and was carried out on a bus, which was close to the traveling conditions using public transportation.

### 2.2. Instruments

#### 2.2.1. Deep Pressure Devices

The devices used to generate deep pressure in this study were the AHMPS with inflatable wrap and manual pull models that have been used in previous studies [29]. The AHMPS is a portable seat made from soft foam covered with synthetic leather fabric which is designed in a shape and size that can be carried and placed on a public bus seat; both models are the same in shape and size. The AHMPS inflatable wrap and manual pull model both have two hugging parts in the form of a transverse strap that is located on the chest and thigh. The difference between the two lies in how the hugging parts exert pressure. In the AHMPS inflatable wraps model, the two hugging parts have a balloon inside which, when exerting pressure, can expand because they are filled with air from the mini pump control system. Meanwhile, in the AHMPS manual pull model, the two hugging parts are transverse foam straps that can be tightened by manually pulling down to exert pressure on the user [27].

#### 2.2.2. Heart Rate (HR) Measurement

Heart rate is one vital sign that is commonly used to measure the physiological effect of deep pressure devices [22,23,30]. Heart rate was measured using Elitech^®^ FOX-1 pulse oximetry (Surabaya, Indonesia) by recording blood oxygenation pulsations. The photoplethysmography (PPG) method is used to detect heart rate variability by emitting or reflecting light rays into the bloodstream; then, the changes in light energy are read as cardiac cycles in relation to systole and diastole [31,32], and PPG signals can be recorded from the finger to calculate reliable heart rate variability estimates [33]. A previous study demonstrated that PPG provides accurate interpulse intervals to measure heart rate variability under ideal conditions [34]. Heart rate is the speed of the heartbeat measured by the number of contractions of the heart/beats per minute (bpm), which represents the average of the peaks of heartbeat waves per minute.

#### 2.2.3. Skin Conductance (SC) Measurement

Skin conductance was measured using galvanic skin response (GSR) [21]. Skin conductance resulting from the variations in the ionic permeability of the sweat gland membrane was captured using a GSR sensor with electrodes that were placed on the fingertips [35]. A custom device with a grove-GSR sensor (Seeed Technology Co., Ltd., Shenzhen, China) was used to measure the skin conductance of all participants. The unit of skin conductance is in micro-siemens (µS), which reflects how much electrical activity is in the skin.

### 2.3. Procedures

Before the experiment was carried out, ethical approval was obtained from the Health Research Ethics Committee Faculty of Medicine, Diponegoro University, number 08/EC/FKUNDIP/I/2019. The consent process was completed prior to the study, and all parents of the 20 children with ASD signed the consent form to participate in this study.

The two AHMPS models were prepared and installed on a medium-sized bus seat in the same row separated by the aisle. The installation of both models was carried out by looping and sticking their horizontal and vertical Velcro straps onto the bus seats. A pretrial session was carried out for all participants for approximately 20 min before engaging in the treatment in order to familiarize them with the two AHMPS models, the pulse oximeter, the GSR device, the presence of researchers, the experimental procedures, and vehicle conditions, and to ensure comfort ahead of the study.

This experimental procedure followed the procedure used in the previous study [27,28], but it was carried out on a moving vehicle, which has never been done before. The study was carried out in the morning at around 9 am in order to avoid physiological and physical fatigue. A teacher (who the children were familiar with) and parent accompanied children with ASD during the study from preparation until the post-test session. The pretest session, called the baseline, was carried out in a regular classroom. The pulse oximetry was placed on the left index finger as a heart rate monitor, and the GSR device was placed on the index and middle fingers of the right hand with Velcro straps to monitor skin conductance. The pretest was registered as a baseline condition before engaging with stressors. The treatment session was carried out on a vehicle during traveling. The air pressure of the AHMPS inflatable wraps model was set at 0.65 psi on the chest and 0.45 psi on the thigh for children in group I, while the pressure of the AHMPS manual pull model was set at 0.81 psi on the chest and 0.80 psi on the thigh for children in group II (determined by placing the load sensor between the hugging parts and the user’s body) [27,28]. Each participant in both groups was administered a treatment session for 20 min on a slow average running speed of less than 40 km/h, running for about 45 min with the same route intended to imitate conditions while traveling by public transportation while measuring heart rate and skin conductance. The treatment session was carried out for 20 min once a week for 3 weeks; thus, each participant received three sessions. The heart rate and skin conductance were recorded in the middle of the session (15 min after departure). Fifteen minutes after the treatment session, the post-test was carried out in a regular classroom, where the pretest was carried out and the heart rate and skin conductance were recorded.

### 2.4. Data Analysis

Data distribution of heart rate variability (HRV) and skin conductance variability was analyzed using the Shapiro–Wilk test; then, a repeated measurement test was applied in order to investigate the distinct sessions and the main effect of deep pressure on physiological responses. A *p*-value < 0.05 was considered statistically significant. The effect of the physiological stress response was reflected in the magnitude of the effect size (η_p_^2^) of the intervention. The value of η_p_^2^ = 0.1 indicated a low effect, η_p_^2^ = 0.3 indicated a medium effect, and η_p_^2^ = 0.5 indicated a high effect. The IBM SPSS software program was used to perform statistical analysis calculations [36].

## 3. Results

Twenty children with ASD (boys: 16, girls: 4) participated in this experimental study, and their ages ranged from 4 to 13 years (mean age was 10.9 ± 2.26 years). All participants were randomly divided into two groups, and each group consisted of 10 children. Group I consisted of nine boys and one girl with the average age of 10.7 ± 2.37 years, while Group II consisted of seven boys and three girls with the average age of 11.1 ± 2.12 years. All participants were Javanese, and there was no significant difference in age (*p* = 0.71) and gender (*p* = 0.28) between the two groups.

The Shapiro–Wilk test was carried out to analyze the data distribution of HRV and skin conductance variability [36] and showed that HRV and skin conductance variability data were normally distributed with *p* > 0.05. The two-way repeated-measures ANOVA test was carried out to analyze the change in physiological anxiety or arousal variable in both groups from baseline/pre-test, on-test, and post-test, i.e., HRV and skin conductance variability. Mauchly’s assumption of sphericity was found not to be violated in HRV (χ^2^ (2) = 2.26 with *p* = 0.322) and skin conductance (χ^2^ (2) = 4.23 with *p* = 0.120), which indicated that the difference in the alteration did not significantly affect the two physiological indicators.

The results of HRV showed that the response within groups and sessions did not display a significant effect, *F* (1, 18) = 1.15, *p* = 0.289 for the groups and *F* (2, 36) = 1.32, *p* = 0.274 for the session. It was concluded that the grouping did not show a significant difference if the sessions were ignored, and vice versa if the grouping was ignored; each session did not show a significant difference between one another.

The results of skin conductance showed the significant effect of deep pressure across groups with the value of *F* (1, 18) = 5.22, *p* = 0.026 and also across session with the value of *F* (2, 36) = 4.68, *p* = 0.013. This indicated that treatments had significant effects on participants based on physiological indicators of skin conductance.

**Figure 1:**
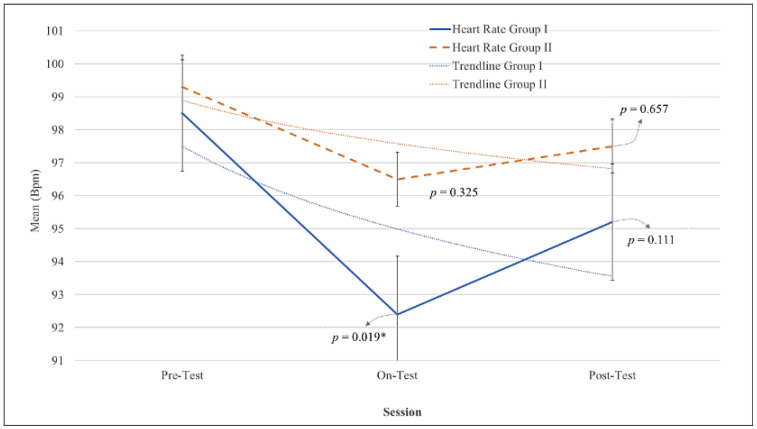
The alterations of heart rate in group I and group II in pre, on, and post-test sessions. Data with * indicated the statistical difference in comparison to the previous session.

**Figure 2:**
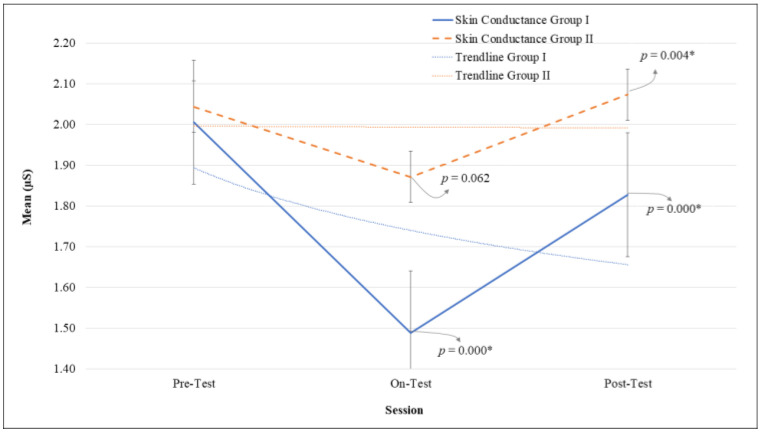
The alterations of skin conductance in group I and group II in pre, on, and post-test sessions. Data with * indicated the statistical difference in comparison to the previous session.

The changes in physiological indicators in both groups during this study are presented in Figure 1 for the HRV and Figure 2 for the skin conductance. Both HRV and skin conductance charts show a similar pattern, which is a decreasing pattern from baseline to treatment, then an increasing pattern from treatment to post-test. The two graphic patterns show that deep pressure treatment using both AHMPS models reduced physiological anxiety levels.

The results of within-subject effects (intragroup data) revealed the comparison between each session. Figure 1 shows the changes in HRV in both groups. In group I, significant changes occurred between the treatment session compared to the baseline (pre-test) session (*p* = 0.019, η_p_^2^ = 0.69), while changes were not found to be significant between the treatment (on-test) session compared to the post-test session (*p* = 0.111, η_p_^2^ = 0.51). In group II, the changes were not found to be significant between sessions, which was the treatment compared to baseline sessions and the treatment compared to post-test sessions. The remaining effect of deep pressure was observed from the trend between the pre-test to post-test session, and the decrease in HRV in both groups was not found to be significant (with *p* = 0.159 dan group 2 with *p* = 0.566).

Figure 2 shows the change in skin conductance value in both groups. The results of group I showed that there were significant changes between the treatment compared to the baseline session with *p* = 0.000 and η_p_^2^ = 0.88, and a similar result was also observed between the treatment compared to the post-test sessions with *p* = 0.000 and η_p_^2^ = 0.87. In group II, the changes were not found to be significant between the treatment compared to the baseline session (*p* = 0.062, η_p_^2^ = 0.58), while the change was significantly different in the treatment compared to the post-test session (*p* = 0.004, η_p_^2^ = 0.79). In order to show the withdrawal effect of deep pressure after the treatment, we analyzed the trend between the two groups, and the remaining effect of deep pressure on skin conductance showed a decreasing trend compared to the baseline condition in group I (*p* = 0.003), but there was no change or a stable trend in group II (*p* = 0.773).

Table 1 presents the mean values of HRV and skin conductance over three weeks of treatment (mean ± SD) for both groups. The changes in the mean value of HRV and skin conductance in both groups displayed the same phenomenon; there was a decreasing pattern from pre-test to on-test and an increasing pattern from on-test to post-test.

The analysis of between-subject effects (intergroup data) was carried out to show that the changes in HRV and skin conductance in each session were due to deep pressure treatment and stressor engagement (taking the bus). The comparison of HRV between sessions in group I and group II was not found to be significant. Meanwhile, the comparison of skin conductance between on-test sessions in group I and group II showed significance with *F* (1, 18) = 6.25, *p* = 0.022, with high effect sizes, η_p_^2^ = 0.507. In addition, the decrease in skin conductance was observed from the baseline/pre-test to the on-test in group I compared to group II; thus, the deep pressure treatment with the AHMPS inflatable wrap model was significantly better compared to deep pressure treatment with the AHMPS manual pull model in this study.

## 4. Discussion

In this study, an evaluation of the effect of deep pressure from two variations of the autism hug machine portable seat (AHMPS) model on ASD children during a trip using public transportation (bus) was measured against physiological indicators such as heart rate and skin conductance. The phenomenon of decreased heart rate (bpm) and skin conductance (µS) indicates that an individual is in a positive emotional state, either relaxed or calm [26]. The three-week treatment sessions of the two AHMPS models used as two group conditions with different participants were analyzed, showing that the intervention session in group I was significantly better than the session in group II regarding the decrease in physiological arousal in children with ASD, observed from both physiological indicators. In other words, the impact of the intervention was positive, especially for group I. The prolonged/remaining effect after the treatment was better in group I both using HRV and skin conductance indicators. Children with ASD in group I showed less physiological arousal rebound compared to children in group II.

In this recent study, the effectiveness of deep pressure application was measured using physiological indicators of heart rate and skin conductance, both of which are part of the physiological stress response [37,38,39]. We found in our study that the skin conductance indicator was closest in capturing autonomic alteration compared to HRV. Bosse et al. used physiological indicators of heart rate and skin conductance in order to assess the impact of video-based stressor induction. The results showed that during the administration of a stressful film, the heart rate indicator did not show a significant difference, while the skin conductance showed a significant difference compared to a non-stressful film [37]. Similar results were also found in a study related to deep pressure application with a short period of application using a weighted blanket [22,23].

Discussion regarding the comparison between the two groups showed that there were significant differences during the stressor and deep pressure application sessions according to skin conductance measurements. The withdrawal effect was shown faster in group II compared to group I; thus, the inflating wrap model had a longer remaining effect of deep pressure compared to the manual pull model. The AHMPS inflatable wrap model in group I reduced physiological arousal significantly better than the AHMPS manual pull model in group II. The purpose of applying deep pressure is to stimulate comfort. However, higher pressure can stimulate discomfort, as might have occurred in group II using the AHMPS manual pull model with higher pressure than group I. The increased skin conductance response was a result of the discomfort stimulation [40,41]. The wider pressure surface also affected the comfort of the deep pressure application [42]. The comfort obtained in group I was provided by a wider pressure surface using the AHMPS inflatable wrap than the manual pull model [27,28]. This supports the findings from the previous study regarding the use of the deep pressure method for the treatment of children with ASD that yielded positive results from its application [20,21,22,23,24,25,26], especially in the context of traveling purposes [27,28].

*Limitations of the study*. The heart rate variability was measured using photoplethysmography (PPG) to capture derived heart rate variability, an indirect HRV in this study, which may be less reliable in not ideal conditions due to motion artifacts. This study was carried out in public transportation, which could have affected the reliability of HRV. Our sample size may have been too small for each group, which may have caused the overestimation of our conclusion, although the magnitude of the experiment was measured and indicated a high effect.

## 5. Conclusions

Deep pressure from the AHPMS decreases the physiological arousal of children with ASD during traveling in public transportation. The inflatable wrap model is more effective in decreasing physiological arousal compared to the manual pull model.

## Figures and Tables

**Table 1:** The mean value of heart rate variability and skin conductance alterations on the pre-test, on-test, and post-test of group I and II.

Sessions	Measures	N	Group I (mean ± SD)	Group II (mean ± SD)	*p*-Value
Pre-test	Heart Rate (bpm)	10	98.5 ± 9.71	99.3 ± 9.476	0.854
	Skin Conductance (µS)	10	2.006 ± 0.4208	2.045 ± 0.3647	0.827
On-test	Heart Rate (bpm)	10	92.4 ± 7.412	96.5 ± 9.675	0.301
	Skin Conductance (µS)	10	1.489 ± 0.3295	1.87 ± 0.3516	0.022 *
Post-test	Heart Rate (bpm)	10	95.2 ± 8.651	97.5 ± 6.654	0.514
	Skin Conductance (µS)	10	1.827 ± 0.3922	2.073 ± 0.3917	0.177

bpm = beats per minute; µS = micro-siemens; N = the amount of sample data; *p* = the significance; * indicates significance.

## Data Availability

The datasets used and/or analyzed during the present study are available from the corresponding author on reasonable request.

## References

[1] American Psychiatric Association (2013). Diagnostic and Statistical Manual of Mental Disorders (DSM-5).

[2] World Health Organization International Statistical Classification of Diseases and Related Health Problems (ICD-11). https://icd.who.int/.

[3] Ministry of Women’s Empowerment and Child Protection of the Republic of Indonesia Hari Peduli Autisme Sedunia: Kenali Gejalanya, Pahami Keadaannya (World Autism Care Day: Recognize the Symptoms, Understand the Conditions). https://www.kemenpppa.go.id/index.php/page/read/31/1682/hari-peduli-autisme-sedunia-kenali-gejalanya-pahami-keadaannya.

[4] Chaste P., Leboyer M. (2012). Autism Risk Factors: Genes, Environment, and Gene-Environment Interactions. Dialogues Clin. Neurosci..

[5] Emberti Gialloreti L., Mazzone L., Benvenuto A., Fasano A., Garcia Alcon A., Kraneveld A., Moavero R., Raz R., Riccio M.P., Siracusano M. (2019). Risk and Protective Environmental Factors Associated with Autism Spectrum Disorder: Evidence-Based Principles and Recommendations. J. Clin. Med..

[6] Leekam S.R., Nieto C., Libby S.J., Wing L., Gould J. (2007). Describing the Sensory Abnormalities of Children and Adults with Autism. J. Autism Dev. Disord..

[7] Chien C.-W., Rodger S., Copley J., Branjerdporn G., Taggart C. (2016). Sensory Processing and Its Relationship with Children’s Daily Life Participation. Phys. Occup. Ther. Pediatr..

[8] Bar-Shalita T., Vatine J., Parush S. (2008). Sensory Modulation Disorder: A Risk Factor for Participation in Daily Life Activities. Dev. Med. Child Neurol..

[9] Yonkman J., Lawler B., Talty J., O’Neil J., Bull M. (2013). Safely Transporting Children with Autism Spectrum Disorder: Evaluation and Intervention. Am. J. Occup. Ther..

[10] Deka D., Feeley C., Lubin A. (2016). Travel Patterns, Needs, and Barriers of Adults with Autism Spectrum Disorder: Report from a Survey. Transp. Res. Rec..

[11] Neo W.X., Flaherty G.T. (2018). Autism Spectrum Disorder and International Travel. Int. J. Travel Med. Glob. Health.

[12] Kohl S.E., Barnett E.D. (2020). What Do We Know about Travel for Children with Special Health Care Needs? A Review of the Literature. Travel Med. Infect. Dis..

[13] McDougle C.J. (2016). Autism Spectrum Disorder.

[14] Samms-Vaughan M.E. (2014). The Status of Early Identification and Early Intervention in Autism Spectrum Disorders in Lower-and Middle-Income Countries. Int. J. Speech. Lang. Pathol..

[15] Matson J.L. (2017). Handbook of Treatments for Autism Spectrum Disorder.

[16] Grandin T. (1992). Calming Effects of Deep Touch Pressure in Patients with Autistic Disorder, College Students, and Animals. J. Child Adolesc. Psychopharmacol..

[17] Taylor C.J., Spriggs A.D., Ault M.J., Flanagan S., Sartini E.C. (2017). A Systematic Review of Weighted Vests with Individuals with Autism Spectrum Disorder. Res. Autism Spectr. Disord..

[18] Ayres A.J. (2013). Bausteine Der Kindlichen Entwicklung: Sensorische Integration Verstehen Und Anwenden-Das Original in Moderner Neuauflage (Building Blocks of Child Development: Understanding and Applying Sensory Integration—The Original in a Modern New Edition).

[19] Ayres A.J. (1972). Sensory Integration and Learning Disorders.

[20] Grandin T. (1984). My Experiences as an Autistic Child and Review of Selected Literature. J. Orthomol. Psychiatry.

[21] Edelson S.M., Edelson M.G., Kerr D.C.R., Grandin T. (1999). Behavioral and Physiological Effects of Deep Pressure on Children with Autism: A Pilot Study Evaluating the Efficacy of Grandin’s Hug Machine. Am. J. Occup. Ther..

[22] Champagne T., Mullen B., Dickson D., Krishnamurty S. (2015). Evaluating the Safety and Effectiveness of the Weighted Blanket with Adults During an Inpatient Mental Health Hospitalization. Occup. Ther. Ment. Health.

[23] Mullen B., Champagne T., Krishnamurty S., Dickson D., Gao R.X. (2008). Exploring the Safety and Therapeutic Effects of Deep Pressure Stimulation Using a Weighted Blanket. Occup. Ther. Ment. Health.

[24] Reynolds S., Lane S.J., Mullen B. (2015). Effects of Deep Pressure Stimulation on Physiological Arousal. Am. J. Occup. Ther..

[25] Chen H.-Y., Yang H., Meng L.-F., Chan P.-Y.S., Yang C.-Y., Chen H.-M. (2016). Effect of Deep Pressure Input on Parasympathetic System in Patients with Wisdom Tooth Surgery. J. Formos. Med. Assoc..

[26] Chen H.-Y., Yang H., Chi H.-J., Chen H.-M. (2019). Parasympathetic Effect of Deep Pressure Input on Third Molar Extraction in Adolescents. J. Formos. Med. Assoc..

[27] Afif I.Y., Farkhan M., Kurdi O., Maula M.I., Ammarullah M.I., Setiyana B., Jamari J., Winarni T.I. (2022). Effect of Short-Term Deep-Pressure Portable Seat on Behavioral and Biological Stress in Children with Autism Spectrum Disorders: A Pilot Study. Bioengineering.

[28] Maula M.I., Aji A.L., Aliyafi M.B., Afif I.Y., Ammarullah M.I., Winarni T.I., Jamari J. (2021). The Subjective Comfort Test of Autism Hug Machine Portable Seat. J. Intellect. Disabil. Treat..

[29] Afif I.Y., Maula M.I., Aliyafi M.B., Aji A.L., Winarni T.I., Jamari J. (2021). Design of Hug Machine Portable Seat for Autistic Children in Public Transport Application. Proceedings of the IOP Conference Series: Materials Science and Engineering.

[30] Becklund A.L., Rapp-McCall L., Nudo J. (2021). Using Weighted Blankets in an Inpatient Mental Health Hospital to Decrease Anxiety. J. Integr. Med..

[31] Moraes J.L., Rocha M.X., Vasconcelos G.G., Vasconcelos Filho J.E., De Albuquerque V.H.C., Alexandria A.R. (2018). Advances in Photopletysmography Signal Analysis for Biomedical Applications. Sensors.

[32] Nardelli M., Vanello N., Galperti G., Greco A., Scilingo E.P. (2020). Assessing the Quality of Heart Rate Variability Estimated from Wrist and Finger PPG: A Novel Approach Based on Cross-Mapping Method. Sensors.

[33] Selvaraj N., Jaryal A., Santhosh J., Deepak K.K., Anand S. (2008). Assessment of Heart Rate Variability Derived from Finger-Tip Photoplethysmography as Compared to Electrocardiography. J. Med. Eng. Technol..

[34] Lu G., Yang F. (2009). Limitations of Oximetry to Measure Heart Rate Variability Measures. Cardiovasc. Eng..

[35] Kim H., Kim Y.S., Mahmood M., Kwon S., Zavanelli N., Kim H.S., Rim Y.S., Epps F., Yeo W.H. (2020). Fully Integrated, Stretchable, Wireless Skin-Conformal Bioelectronics for Continuous Stress Monitoring in Daily Life. Adv. Sci..

[36] Field A. (2013). Discovering Statistics Using IBM SPSS Statistics.

[37] Bosse T., Gerritsen C., de Man J., Stam M. (2014). Inducing Anxiety through Video Material. Commun. Comput. Inf. Sci..

[38] Epstein S., Roupenian A. (1970). Heart Rate and Skin Conductance during Experimentally Induced Anxiety: The Effect of Uncertainty about Receiving a Noxious Stimulus. J. Pers. Soc. Psychol..

[39] Lin H.P., Lin H.Y., Lin W.L., Huang A.C.W. (2011). Effects of Stress, Depression, and Their Interaction on Heart Rate, Skin Conductance, Finger Temperature, and Respiratory Rate: Sympathetic-Parasympathetic Hypothesis of Stress and Depression. J. Clin. Psychol..

[40] Hein G., Lamm C., Brodbeck C., Singer T. (2011). Skin Conductance Response to the Pain of Others Predicts Later Costly Helping. PLoS ONE.

[41] Awad A.A., Ghobashy M.A.M., Ouda W., Stout R.G., Silverman D.G., Shelley K.H. (2001). Different Responses of Ear and Finger Pulse Oximeter Wave Form to Cold Pressor Test. Anesth. Analg..

[42] Minoura M., Tani I., Ishii T., Gunji Y.-P. (2019). Observing the Transformation of Bodily Self-Consciousness in the Squeeze-Machine Experiment. JoVE J. Vis. Exp..

